# Administration of the Postbiotic Substance 412 to Slovak Warm-Blooded Horses: A Pilot Experiment

**DOI:** 10.3390/life16081342

**Published:** 2026-08-16

**Authors:** Andrea Lauková, Valentína Focková, Lenka Micenková, Soňa Gancarčíková, Iveta Plachá, Ľubomíra Grešáková, Anton Kováčik, Marko Halo, Branislav Gálik, Monika Pogány Simonová

**Affiliations:** 1 Center of Biosciences of the Slovak Academy of Sciences, Institute of Animal Physiology, Šoltésovej 4-6, 040 01 Košice, Slovakia; valentina.fockova@gmail.com (V.F.); placha@saske.sk (I.P.); gresakl@saske.sk (Ľ.G.); simonova@saske.sk (M.P.S.); 2 Department of Experimental Biology, Faculty of Science, Masaryk University, Kotlářska 2, 611 37 Brno, Czech Republic; micenkova.lenka@gmail.com; 3 Department of Microbiology and Immunology, University of Veterinary Medicine and Pharmacy, Komenského 73, 041 83 Košice, Slovakia; sona.gancarcikova@uvlf.sk; 4 Institute of Applied Biology, Faculty of Biotechnology and Food Sciences, Slovak University of Agriculture in Nitra, Tr. A. Hlinku 2, 949 76 Nitra, Slovakia; anton.kovacik@uniag.sk; 5 Institute of Animal Husbandry, Faculty of Agrobiology and Food Resources, Slovak University of Agriculture in Nitra, Tr. A. Hlinku 2, 949 76 Nitra, Slovakia; marko.halo@uniag.sk; 6 Institute of Nutrition and Genomics, Faculty of Agrobiology and Food Resources, Slovak University of Agriculture in Nitra, Tr. A. Hlinku 2, 949 76 Nitra, Slovakia; branislav.galik@uniag.sk

**Keywords:** enterocin-like substance, effect, horse, health, metabolic parameters

## Abstract

Antimicrobial proteinaceous postbiotic substances (PS) and/or bacteriocins can beneficially influence animal health, as previously shown with enterocins from non-autochthonous enterococcal strains. Therefore, the effectiveness of PS 412, produced by our beneficial autochthonous strain *Enterococcus faecium* EF 412, was assessed in horses. In the pilot experiment, PS 412 was administered to 12 horses of various ages and breeds for 21 days via a small feed ball. Sampling was performed on days 0/1, 21, and 42 (after a 3-week cessation of PS 412). The horses served as their own controls. Although the number of horses was limited, the microbiological analysis showed significant decreases in enterococci, other lactic acid bacteria, and campylobacters (*p* < 0.05; *p* < 0.001). Staphylococci decreased slightly. Coliforms, aeromonads (*p* < 0.001), and pseudomonads also decreased. In next-generation sequencing at the phylum level, 15 phyla were detected at higher abundance, with Bacteroidota (35.38%) and Bacillota (32.25%) the most abundant. After PS 412 (activity 51,200 AU/mL), the relative abundance of several phyla decreased. On day 21, phagocytic activity was higher (69.00 ± 3.91%). The metabolic profiles and hematologic values were mostly within the reference range. Although continued testing is necessary, postbiotics appear to be a tool for supporting equine health.

## 1. Introduction

Beneficial (probiotic) bacteria are widely used as nutritional supplements and treatment interventions in the management of livestock and companion animals [1]. For instance, the immune response of the beneficial non-autochthonous strain *Enterococcus faecium* AL 41 (CCM 8558, accession number in GenBank (AN) MW256492) was noted in chicks experimentally infected with *Campylobacter jejuni* CCM 6191. The strain AL 41 predominantly acted 24 h *post*-infection with *C. jejuni* [2]. It displayed a distinct pattern of T- and B- cell activation. For example, in vivo effects and antimicrobial activity in broiler rabbits were observed in feces and caecum after the application of the *Enterococcus faecium* AL 41 strain (CCM 8558) and its Enterocin M (Ent M, a highly purified postbiotic substance). Coliforms were significantly reduced (*p* < 0.05), as were pseudomonads, and *Staphylococcus aureus* [3]. Despite the fact that the use of probiotics in horses is rather rare, *E. faecium* AL 41 demonstrated an increasing tendency in phagocytic activity (PA) after 14 days of its application. Hydrolytic activity was also significantly increased (*p* < 0.001) [4]. Moreover, the application of the autochthonous beneficial strain *E. faecium* EF 412 (GenBank accession MW256494) in Slovak warm-blooded horses tended to result in higher values of PA. There was also a decrease in serum cholesterol and triglycerides on day 21 compared with day 0/1 and day 35 (2 weeks after cessation of the strain) [5]. The beneficial strains mentioned produce postbiotic substances of proteinaceous nature (bacteriocins-enterocins) that are antimicrobially active [5,6,7,8]. Although the use of postbiotic substances in horse breeding and/or diet is not frequent, as reported in our previous study, administration of Ent M led to a significant reduction in coliforms, campylobacters, and *Clostridium* spp. (*p* < 0.001). An increase in PA values was also observed on day 21 (*p* < 0.05) and day 42 (*p* < 0.001), indicating a prolonged beneficial effect of Ent M [8]. The last-mentioned strain *E. faecium* EF 412 is susceptible to antibiotics and exhibits sufficient adhesion to human and canine mucus. It possesses genes for the production of Enterocins A and B. The antimicrobial proteinaceous substance produced by the strain EF 412 is thermostable [9]. It has not yet been purified to homogeneity. According to current knowledge, non-purified antimicrobial substances of proteinaceous origin are classified as a group of postbiotics. They are defined as preparations of inanimate microbiota and/or their components that confer a health benefit to the host [10,11]. The antimicrobial active proteinaceous substances purified to homogeneity are bacteriocins [11].

Probiotics and postbiotics play an important role in intestinal health. Compared with probiotics, postbiotics offer several advantages; for example, they can act not only in the intestinal tract but also in the oral cavity, skin, and other tissues [12]. They are heat-stable and acid-resistant. Because they are not alive, they cannot transfer virulence factor genes, making them safe. Mosca et al. [13] reported that postbiotic substances can improve the host’s immune function and, in turn, influence, for example, the host’s diarrhea status. Given the limited scope, we administered the postbiotic substance (PS 412) produced by the strain *E. faecium* EF 412 to Slovak warm-blooded horses to assess its effects on the microbiota, PA, glutathione peroxidase (GPx), serum biochemistry, and/or blood parameters. Taken together, this supports the hypothesis that postbiotics would serve as a new tool to support the health of breeding horses. To underscore our aim, the novelty of testing PS 412 lies in the fact that to date, only its producing strain has been tested in horses, and this substance is produced by the autochthonous (horse-derived) species strain *Enterococcus faecium* EF 412.

## 2. Materials and Methods

### 2.1. Ethical Approval

All procedures performed in the experiment were approved by the Ethical Committee of the Institute of Animal Physiology, Center of Biosciences of the Slovak Academy of Sciences in Košice (Slovakia, Apr. no. 339004). All manipulations were in accordance with standard veterinary practices as defined in Slovak legislation (nos. 377/2012, 436/2012). The horse owners agreed to participate in this study.

### 2.2. Preparation of Postbiotic Substance (Enterocin-like) 412 for Application

PS 412 is produced by the autochthonous strain *Enterococcus faecium* EF 412, which is deposited in GenBank under accession number MW256494. For application purposes, the substance was prepared according to the protocol for the dipeptide Enterocin A/P reported by Mareková et al. [14] with modifications. Briefly, 300 mL of MRS broth (de Man-Rogosa–Sharpe broth, pH 7.1, Merck, Darmstadt, Germany) was inoculated with an overnight 0.1% pre-culture of the producing strain *E. faecium* EF 412. The inoculated MRS broth (containing strain EF 412) was incubated at 37 °C overnight until the absorbance at 600 nm (A_600_) reached 1.0. The broth culture was centrifuged at 10,000× *g* for 30 min. The supernatant (pH 5.0) was precipitated with ammonium sulfate (40% saturation) at 4 °C for 4 h. After centrifugation (10,000× *g* for half an hour), the precipitate was re-suspended in a minimal volume of 10 mM phosphate buffer (pH 6.5). Activity of the precipitate was tested using the agar spot test [15]. Active postbiotic substance PS 412 exhibited an inhibitory activity of 51,200 AU/mL against the principal indicator strain *Enterococcus avium* EA5 (our strain).

### 2.3. Horses and Experimental Design

Twelve clinically healthy horses (six mares and six stallions of the Slovak warm-blooded breed) were included in this experiment. They were owned by private clients who agreed to participate. Because they are used for riding, only a limited number of horses were accepted for experimentation. The horses were housed in the stable (CE SK 339004) of the Riding Center of the Slovak Agricultural University (SAU) in Nitra (Slovakia) under the supervision of Marko Halo, in accordance with the Guide for Animal Practice approved by the Ethics Committees of the participating institutes in the experiment (Apr. No. 339004) as previously indicated by Lauková et al. [5]. The horses were placed in separate boxes. They were aged as previously reported by Lauková et al. [5]: a 12-year-old stallion (no. 1), an 8-year-old mare (no. 2), an 11-year-old mare (no. 3), a 12-year-old mare (no. 4), a 5-year-old horse (no. 5), a 7-year-old mare (no. 6), a 5-year old horse (no. 7), a 5-year-old mare (no. 8), a mare aged 10 years (no. 9), a 8-year-old horse (no. 10), a 9-year -old horse (no. 11), and a horse aged 13 years (no. 12). They were fed hay (10 kg per animal) and whole oats (2 to 4 kg) or grazed. They had *ad libitum* access to water (via automatic nipples). They were engaged in light or moderate work/exercise. The experiment lasted 42 days. Sampling was performed at the start of the experiment (day 0/1), on day 21 (after a 3-week application of PS 412), and at the end of the experiment (day 42, after a 3-week cessation of PS 412). Feces were collected in the morning after each animal defecated. Blood was sampled from the *vena jugularis.* Fecal samples (*n* = 12) were immediately stored in a transport fridge box (4 °C). Fecal samples for next-generation sequencing analysis (NGS) were frozen. After transport to the laboratory, they were analyzed, treated, and stored for subsequent analyses. Each horse served as its own control for each sampling. Its status at the start of the experiment (day 0/1) was compared with its status after application of PS 412 (day 21, after a 3-week application) and after its cessation (day 42). After initial sampling, the animals were administered PS 412 (100 µL) in a small feed ball to ensure that the specified horse consumed it. PS 412 was applied for 3 weeks (21 days). The animals followed a standard drinking regimen. After the experiment, they were used by clients and stabled at SAU, Nitra (Slovakia).

### 2.4. Standard Microbial Testing

Feces (1 g) from each horse (*n* = 12) were diluted using the standard microbiological method (Ringer solution, 1:9, Merck, Darmstadt, Germany, pH 7.0), and then homogenized with a Stomacher IUL (Instruments, Barcelona, Spain) in accordance with the International Organization for Standardization (ISO). 100 µL of the appropriate dilution was plated on M-Enterococcus agar (ISO 7889, Difco, Detroit, MI, USA) to enumerate total enterococci. De Man–Rogosa–Sharpe agar (1.5%, MRS, Merck, Darmstadt, Germany) was used to enumerate other lactic acid bacteria (LAB). Baird-Parker agar supplemented with egg yolk tellurite solution (ISO 21527-1, Difco) was used to enumerate coagulase-positive staphylococci (CoPS). Coagulase-negative staphylococci (CoNS) were enumerated on Mannitol salt agar (Difco). Clostridium (Clostridioides) difficile agar with SR0096E supplement and 7% (*v*/*v*) defibrinated horse blood (SR0050, ISO 15883, Oxoid Ltd., Basingstoke, Hampshire, UK) was used to detect *Clostridium* spp. Coliforms were detected on MacConkey agar (ISO 7402, Difco). *Aeromonas* spp. were counted using Aeromonas (Ryan) agar (Oxoid). Plates were incubated at 37 °C for 24–48 h. *Pseudomonas* spp. were enumerated on Pseudomonas agar (Biomark, India) after incubation at 30 °C for 24 h. Campylobacters were isolated on the Campylobacter selective medium, Karmali, with supplement CM0935 (Oxoid, Waltham, Massachusetts, USA). In this case, cultivation was performed at 42 °C for 42–48 h. Bacterial counts were expressed in (log 10) colony-forming units per gram (CFU/g) ± SD.

### 2.5. Microbiome Analysis Using Next-Generation Sequencing

The protocol used for this analysis was reported in our previous study [5]. The primers used are summarized in Table 1. Briefly, isolated DNA (according to the manufacturer’s protocol) using a PowerLyzerR PowerSoilR isolation kit (Qiagen, Hilden, Germany) was used as a template in a PCR reaction targeting the hypervariable V3-V4 region (341F-785R) of the bacterial 16S rRNA gene (16S Metagenomic Sequencing Library Preparation protocol, Illumina Way, San Diego, California, USA, Table 1). Sequencing was performed using MiSeq reagent Kits v3 on a MiSeq 2000 sequencer according to manufacturer’s instructions (Illumina). Sequences were analyzed using the workflow provided by QIIME 1.9.1. [16]. Quality trimming using the Trimmomatic version of the software v0.39 [17] was performed as part of sequence processing, followed by demultiplexing (a house tool written in Python 3) and joining [18]. Operational taxonomic units (OTUs) were constructed by clustering sequences at a 97% similarity threshold and selecting the representative sequences of clusters as OTUs. Clustering was conducted using the UCLUST algorithm [19]. Taxonomy was assigned to each OTU using the USEARCH LCA algorithm, which is based on the SILVA 123 reference database [20]. Re-classified phyla names are included in Int. J. Syst. Evol. Microbiol., 2021, 71, 005056 [21].

### 2.6. Phagocytic Activity, Blood and Serum Parameters

Blood (8 tubes from each horse) was collected into Eppendorf tubes containing microspherical hydrophilic particles (MSHP) and heparin. Direct counting was performed. Ingestion of MSHP by polymorphonuclear cells (PMN) was determined as follows: 50 μL of SH particle suspension (ARTIM, Prague, Czech Republic) was mixed with 100 μL of blood in an Eppendorf-type tube and incubated at 37 °C for one h. Blood smears were stained using the May–Grünwald and Giemsa–Romanowski system. Phagocytic activity (PA) was calculated as the number of white cells containing at least three engulfed particles per 100 white cells (neutrophils and monocytes). The percentage of phagocytic cells was evaluated under an optical microscope by counting up to 100 PMN. Subsequently, the PA index (IPA) was calculated.

Regarding serum chemistry in horses (8 tubes from each horse), the following profiles were analyzed: nitrogen profile (total proteins g/L, albumin g/L, urea mmol/L, creatinine µmol/L); energy profile (triglycerides and cholesterol in mmol/L); enzymatic and hepatic profile (AST, aspartate-aminotransferase, µkat/L; ALT, alanine-aminotransferase, µkat/L, ALP, alkaline phosphatase, µkat/L, GMT (GGT, ɤ-glutamyl transferase, µkat/L; total bilirubin, µmol/L); CK, creatine kinase, µkat/L; GLDH, glutamate dehydrogenase, µkat/L; LDH-L, lactate dehydrogenase, µkat/L.; and mineral profile (Ca, calcium mmol/L; P, phosphorus; Mg, magnesium, and chlorides in mmol/L). Testing was performed using the DiaSys kit (Diagnostic Systems GmbH, Holzheim, Germany) or the commercial DIALAB tests (Prague-Radotín, Czech Republic) with the Ellipse analyzer (AMS, Guidonia, Italy). Minerals were analyzed using an EasyLite analyzer via ion-selective electrode [22]. Hematological parameters (8 tubes from each horse) were analyzed using the Abacus Vet 5 analyzer (Diatron, Budapest, Hungary) as previously described [23].

### 2.7. Statistical Assessment

Treatment effects on fecal microbiota (assessed using the standard method), phagocytic activity (PA), GPx, serum chemistry, and hematologic values were analyzed using one-way analysis of variance (ANOVA), with Tukey’s post hoc test, where appropriate. This is an acceptable limitation in this type of evaluation. Data are presented as means and standard deviation of the mean (SD) and were compared among groups on the same days of sample collection to assess changes during the experiment. Differences in mean values across treatments were considered statistically significant at *p* < 0.05. Analyses were performed using GraphPad Prism (version 6.0, GraphPad Software, San Diego, CA, USA). NGS results were evaluated as relative abundance percentages.

## 3. Results

### 3.1. Microbiota Determination Before PS 412 Application, on Day 21 (After a 3-Week Application) and 42 (After a 3-Week Cessation) Using Standard Microbiological Method

Using the standard microbiological method, microbiota counts were most affected (Table 2a). Regarding Gram-positive bacteria, enterococci were significantly reduced on day 21, after a 3-week application (*p* < 0.05), followed by an increase on day 42; however, the count remained lower than at the start of the experiment. The count of LAB also decreased (*p* < 0.01) compared to day 0/1 and days 21 and 42 (Table 2a). Staphylococci (CoPS, CoNS) were not affected; however, in CoPS, a slight difference (0.54, 0.74 log cycles) was observed between day 0/1 and days 21 and 42 (Table 2a). Clostridiae were affected; however, their counts were higher on days 21 and 42. For the Gram-negative genera, Campylobacters showed a significant decrease (Table 2b, *p* < 0.001) when comparing day 0/1 with day 42, and also between days 21 and 42 (*p* < 0.01). Pseudomonads were not significantly affected; only slight decreases with differences of 0.67 and 1.01 cycles were observed on day 21 compared to days 0/1 and 42. Aeromonads were significantly reduced (*p* < 0.01) on days 21 and 42 compared to day 0/1. Similarly, coliforms were significantly reduced on day 21 compared to days 0/1 (*p* < 0.001) and 42 (*p* < 0.001; Table 2b).

### 3.2. Next-Generation Sequencing Results

**Figure 1:**
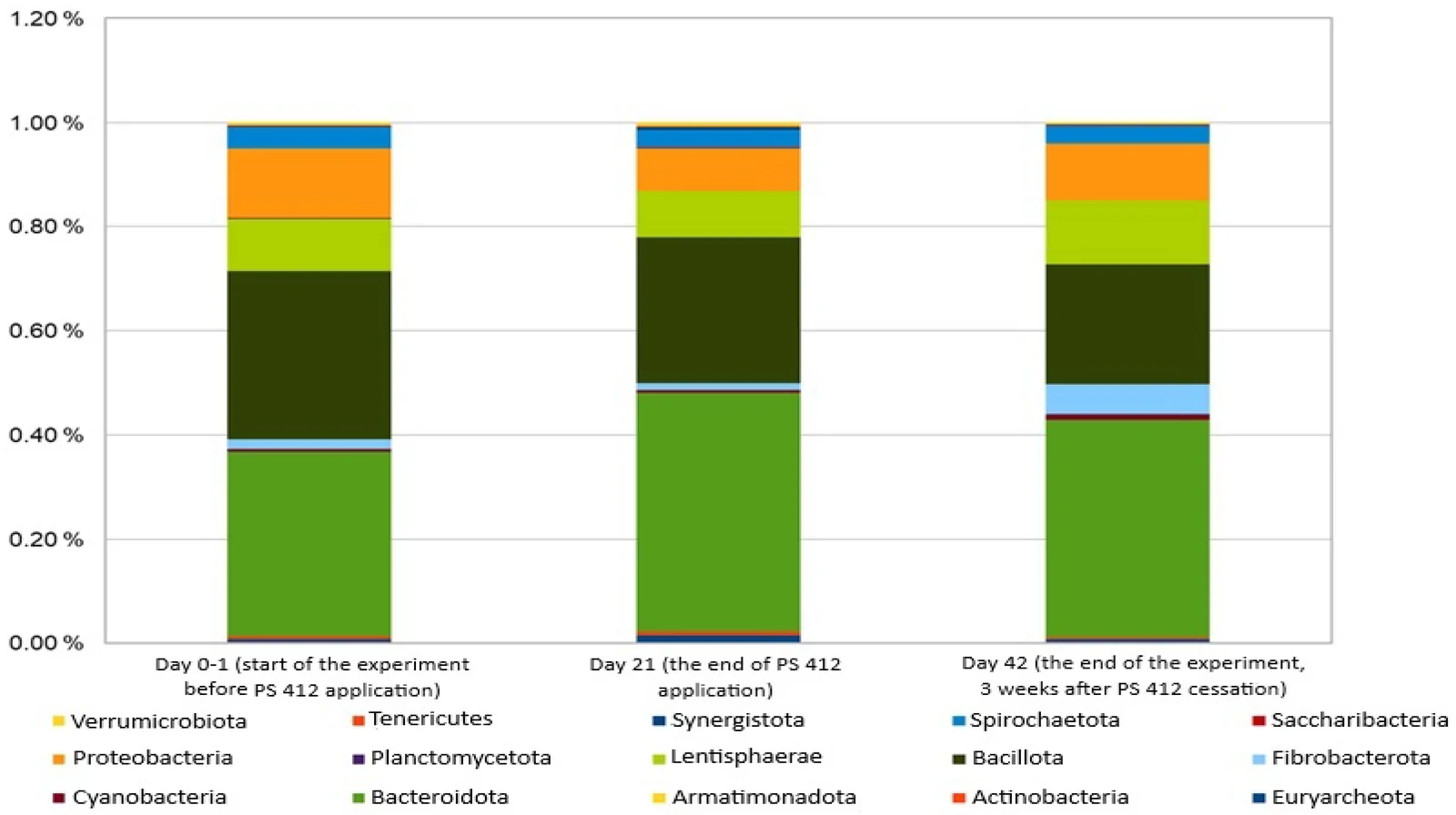
Microbiota tested using next-generation sequencing at the phylum level (percentage of abundance). Bacillota (previously Firmicutes), Bacteroidota (previously Bacteroidetes), Fibrobacterota (previously Fibrobacteres), Spirochaetota (previously Spirochaetae), Verrumicrobiota (previously Verrumicrobia), Synergistota (previously Synergistetes), Armatimonadetes Armatimonadota (previously Armatimonadetes), Planctomycetota (Planctomycetes): Oren and Garrity, *Int. J. Syst. Evol. Microbiol.*, 2021, 71, 005056 [21].

NGS results are reported at the phylum level. Fifteen phyla were detected, with high relative abundances (%, Table 3). The highest relative abundance was observed for the phylum Bacteroidota (35.38%), followed by Bacillota (32.25%, Table 3, Figure 1), Proteobacteria (13.36%), Lentisphaera (10.03%), Spirochaetota (4.01%), and Fibrobacterota (2.01%). For the phyla Armatimonadetes, Planctomycetes, Saccharibacteria, Synergistota, Tenericutes, Verrumicrobiota, Cyanobacteria, Actinobacteria, and Euryarcheota, relative abundance ranged from 0.05 to 0.65% (Table 3). Application of PS 412 on day 21 probably contributed to decreases in Bacillota (28.10%), Proteobacteria (8.19%), Lentisphaera (8.83%), Spirochaetota (3.34%), Cyanobacteria (0.47%), Tenericutes (0.16%), and Fibrobacterota (1.32%; Table 3), while relative abundance in Euryarcheota, Actinobacteria, Verrumicrobiota, Saccharibacteria, Planctomycetota, and Armatimonadota was slightly (numerically) increased. On day 42 (after a 3-week cessation of PS 412), Bacteroidota decreased, and relative abundances of the phyla Euryarcheota, Actinobacteria, Verrumicrobiota, Synergistota, and Saccharibacteria decreased. The other phyla were unaffected; they remained at almost the same levels as on day 21. On the other hand, increases in the phyla Fibrobacterota (Fibrobacteres), Proteobacteria, and Lentisphaerae were observed (Table 3).

### 3.3. Results of Phagocytic Activity, Blood, and Serum Chemistry

The PA and IPA values were well-balanced (Table 4); however, on day 21, PA showed a non-significant (*p* = 0.3762) but numerically increasing trend (Table 4; increase from 67.59 ± 3.66% on day 0/1 to 69.00 ± 3.91% on day 21). On day 42, PA values returned to the same value as measured on day 0/1 (Table 4). GPx was not significantly decreased (Table 4); however, it showed well-balanced values, with only a slight change on day 21 (Table 4).

Regarding the enzymatic and hepatic profile in horse serum, AST values were significantly lower on day 21 (*p* < 0.05, Table 5) than on day 0/1 and day 42. The AST values on day 0/1 and after a 3-week cessation of PS 412 were nearly identical. However, during the experiment, they approached the upper limit of the AST reference value (Table 5). After PS 412 administration, the ALT value showed a beneficial decrease (differences of 0.82 and 0.95) on day 21 compared with day 0/1 and day 42 (Table 5). The values measured during the experiment were within the physiological (reference) range reported for horses. A decrease in the enzyme ALP was also noted, with a prolonged decline (Table 5), although the values remained within the reference range. GMT/GGT values were also within the reference range and decreased slightly after administration of PS 412 (Table 5). LDH-L activity was significantly reduced on day 21 (*p* < 0.001) compared with day 0/1 and on day 42 (*p* < 0.05; Table 5). The GLDH values were slightly elevated, but remained within the reference range (Table 5). The CK values were within the reference range; however, after application of PS 412 (day 21), they decreased by a numerical difference of 1.22, then increased to nearly the same value as on day 0/1 (Table 5). Moreover, total bilirubin decreased slightly (numerically) and remained within the reference range. Regarding the energy profile, HDL cholesterol decreased slightly, whereas LDL cholesterol was significantly lower on day 21 than on day 0/1 (*p* < 0.01). However, both remained above the reference range (Table 6). The parameters in the nitrogen profile were well balanced, as were albumin and urea values (Table 7). Creatinine values were elevated but remained within the reference range. The mineral profile showed a decrease in P and a slight decrease in Ca compared with day 0/1 and day 42. A significant decrease in Mg was observed on day 42 (*p* < 0.001), with a slight increase on day 21. Chlorides increased significantly on day 21 (*p* < 0.01), followed by a decrease to the level observed on day 0/1 (Table 7).

Regarding the hematologic parameters (Table 8), most values remained within the reference range. The mean count of erythrocytes (MCV) was slightly increased but remained within the reference range. Thrombocytes were increased (100.0) on day 21 and were below the reference limit of 74.00.

## 4. Discussion

The advantage of postbiotics lies in their great potential to maintain intestinal homeostasis and health by retarding the growth of intestinal pathogens [24]. They help to reduce bacterial numbers, especially those of unwanted bacteria [10]. It was reported that a bacterial postbiotic derived from the commercially used probiotic strain *Lactobacillus rhamnosus* GG could prevent damage to human intestinal smooth muscle cells caused by intestinal pathogens [25]. Although only a limited number of horses were included, the application of PS 412 in horses influenced the microbiota. Enterococci were significantly decreased (*p* < 0.05), and the count of other LAB was also decreased (*p* < 0.05) using the standard microbiological method for their evaluation. Enterococci are generally susceptible to PS or bacteriocins produced by enterococci, as closely related bacteria. The same explanation could be used to explain LAB reduction. The count of staphylococci was unaffected or decreased slightly. Although staphylococci are lactic acid bacteria, their cell membranes are less susceptible to damage, and they can exhibit resistance to PS depending on the appropriate PS’s mode of action. Among Gram-negative bacteria, campylobacters were significantly decreased (*p* < 0.001), as were coliforms and aeromonads (*p* < 0.001). A decrease in pseudomonads was also observed. Similar results were reported in our previous study, in which the postbiotic substance purified as Ent M was applied to horses. After its application, a significant reduction in campylobacters (*p* < 0.05) and clostridiae (*p* < 0.001) was noted, and a reduction in coliforms as well [8]. In our application experiment, using semi-purified PS 412 in horses, the results indicated that it likely acts as a class II bacteriocin-enterocin, a small thermostable peptide, generally known for its broad antimicrobial spectrum [6,7]. The reduction in campylobacters and other bacteria by postbiotic substances could be explained by several hypotheses. Several mechanisms exist; for example, PS can bind to specific targets on the lipid bilayer of susceptible bacteria, creating the proton-motive force. They can also disrupt bacterial cell membranes, forming pores and causing lethal leakage of ions and cellular content. It could be supposed that in the case of pseudomonads, they could disrupt the integrity of existing biofilms (formed by pseudomonads) and prevent their formation. As previously reported, Ent M (PS purified to homogeneity but used in semi-purified form in the experiment) showed a similar antimicrobial effect in horses and belongs to the same II class of bacteriocins. Conversely, the lack of effectiveness against clostridiae can probably be explained by their resistance to PS 412. Regarding next-generation sequencing results based on phyla analysis, Bacteroidota and Bacillota were detected in the highest relative abundance in the feces of horses after application of PS 412, which correlates with findings of Dougal et al. [26]. There, the % abundance was almost the same for Bacillota as in our study (32.25% and 28.10%, respectively). They also detected Spirochaetota (previously Spirochaeta), Fibrobacterota, and Actinobacteria at % of abundance of <4% [26]. Although limited analysis was presented in this study based on NGS (only the category phylum, not the categories order, class, family, and genus), and a limited number of horses as well, this analysis contributes to understanding horses’ microbial status in the case of PS application and cessation. The application of the postbiotic substance is advantageous because its use does not require a special safety declaration, as is required in the case of probiotic/beneficial bacteria [27]. To date, in our case, postbiotics have been administered to healthy horses; however, their application and effects in diseased horses could underscore their antimicrobial efficacy and their stimulation of non-specific immune parameters, such as phagocytic activity. In our study, PA tended to be higher after the application of PS 412. This effect on PA was also previously noted after the application of PS and/or Ent M in horses (*p* < 0.05; *p* < 0.001; [8]. As with PS 412 in this study, for example, the application of *E. faecium* EF 412 in horses (which produces PS 412) tends to increase PA [5]. Based on the results, PS 412 did not affect oxidative stress in horses. Application of PS 412 had a beneficial effect on selected parameters of the enzymatic and hepatic profile. Compared with day 0/1, AST decreased (*p* < 0.05). Significantly lower LDH values (*p* < 0.001) were observed. PS can act by targeting the asialoglycoprotein receptor (ASGR1) in the host’s liver. This action triggers a process that reduces lipid and/or fat storage. PS can bind to ASGR1 in liver cells, leading to its proteasomal degradation. There were also lower levels of the enzymes ALT, GMT/GGT, ALP, and creatine kinase, as well as total bilirubin, and a significant decrease in LDL-cholesterol (*p* < 0.01). In the nitrogen profile, there was no supporting effect, but in the mineral profile, Ca and Mg increased. Minerals play a critical role in horse health. Higher Ca is a useful parameter, and Mg utilization decreases with age. Therefore, the horse’s age is important for this parameter [28]. A decrease in total protein indicates reduced protein synthesis. Hisaeda et al. [29] reported that season can influence mineral concentrations and other parameters in horses’ serum. They found that creatinine, total protein, P, and Mg were higher in summer, whereas Ca, chloride, albumin, cholesterol, hemoglobin, and hematocrit were lower. Our sampling was conducted in the spring, so we found some values similar and others that differed. The results obtained represent a new contribution to the limited research published to date on the efficacy and tolerability of postbiotics in equids [1]. Among them, for example, Terpeluk et al. [30] mentioned SCFP-postbiotics (*Saccharomyces cerevisiae* fermentation product), a cultured yeast product, eco-friendly, and non-antibiotic, showing a stable microbial profile in horses after stress challenge. A diet supplemented with an SCFP affects the immediate response to parenteral vaccination against influenza and tetanus in the early life of horses [31]. It resulted in increased numbers of granulocytes, lymphocytes, monocytes, CD4+ T cells, CD8+ T cells, CD21+ B cells, and other immune cells [32]. Moreover, studies by Kraft et al. [33] suggest that postbiotic supplementation in horses may influence their immune response to vaccination. Our results support the hypothesis that postbiotics, as multifunctional microbial products, can support animal health and performance [21].

## 5. Conclusions

Although only a limited number of horses were used in the pilot experiment, it can be assumed that administering PS 412 in a small feed ball to horses for 21 days (activity 51,200 AU/mL) caused a significant decrease in enterococci and LAB based on the standard microbiological method. In addition, a decrease in selected Gram-negative bacteria was noted. The PS application contributed to a decrease in the relative abundance of several bacterial phyla. After a 3-week application of PS 412, PA tended to be higher. The PS application did not induce oxidative stress. Most biochemical parameters remained within reference ranges. Despite the limited experimentation, postbiotics may be a potential tool for supporting health in horse breeding, although ongoing testing is expected.

## Figures and Tables

**Table 1:** List of primers. 16S Metagenomic Sequencing Library Preparation protocol (Illumina, USA) (EMP 515-806). Previously introduced in the article by Lauková et al. [5].

Gene	Primer Locus Sequence
EMP16S-1	F:5′-TCGTCGGCAGCGTCAGATGTGTATAAGAGA-3′ 5′CAGAGCCTTCGTCGCGTGTGYCAGCMGCCGCGGTAA-3′
	R:5′-GTCTCGTGGGCTCGGAGATGTGTATAAGAGACAGCCTAAC-3′ 5′GGTCCACCGGACTACNVGGGTWTCTAAT--3′
EMP16S-2	F:5′-TCGTCGGCAGCGTCAGATGTGTATAAGAGACAGTCCATACCG-3′ 5′-GAAGTGTGYCAGCMGCCGCGGTAA-3′
	R:5′-GTCTCGTGGGCTCGGAGATGTGTATAAGAGA-3′
	5′-CAGCGCGCCTTAAACCCGGACTACNVGGGTWTCTAAT-3′
EMP16S-3	F:5′-TCGTCGGCAGCGTCAGATGTGTATAAGAGACAGAGCCCTGC-3′
	5′-TACAGTGTGYCAGCMGCCGCGGTAA-3′
	R: 5′-GTCTCGTGGGCTCGGAGATGTGTATAAGAGACAGTATGG-3′ 5′-TACCCAGCCGGACTACNVGGGTWTCTAAT-3′
EMP16S-4	F:5′-TCGTCGGCAGCGTCAGATGTGTATAAGAGACAGTGA-3′ 5′-GACCCTACAGTGTGYCAGCMGCCGCGGTAA-3′
	R:5′-GTCTCGTGGGCTCGGAGATGTGTATAAGAGACAGGCCTCTAC-3′ 5′-GTCGCCGGACTACNVGGGTWTCTAAT-3′
EMP16S-5	F:5′-TCGTCGGCAGCGTCAGATGTGTATAAGAGACAGACTTGGTG-3′ 5′-TAAGGTGTGYCAGCMGCCGCGGTAA-3′
	R: 5-GTCTCGTGGGCTCGGAGATGTGTATAAGAGACAGACTACTGAG-3′ 5′-GATCCGGACTACNVGGGTWTCTAAT-3′
EMP16S-6	F:5′-TCGTCGGCAGCGTCAGATGTGTATAAGAGACAGATTAC-3′ 5′-GTATCATGTGTGYCAGCMGCCGCGGTAA-3′
	R:5′-GTCTCGTGGGCTCGGAGATGTGTATAAGAGACAGAATTCAC-3′ 5′-CTCCTCCGGACTACNVGGGTWTCTAAT-3′
EMP16S-7	F: 5′-TCGTCGGCAGCGTCAGATGTGTATAAGAGACAGCACGCAGTC TAC-3′ 5′-GTGTGYCAGCMGCCGCGGTAA-3′
	R:5′-GTCTCGTGGGCTCGGAGATGTGTATAAGAGACAGCG-3′ 5′-TATAAATGCGCCGGACTACNVGGGTWTCTAAT-3′
EMP16S-8	F:5′-TCGTCGGCAGCGTCAGATGTGTATAAGAGACAGTGTGCAC-3′ 5′-GCCATGTGTGYCAGCMGCCGCGGTAA-3′
	R:5′-TCTCGTGGGCTCGGAGATGTGTATAAGAGACAGATGCTG-3′ 5′-CAACACCCGGACTACNVGGGTWTCTAAT-3′
EMP16S-9	F:5′-TCGTCGGCAGCGTCAGATGTGTATAAGAGACAGCCGGACAA-3′ 5′-GAAGGTGTGYCAGCMGCCGCGGTAA-3′
	R:5′-GTCTCGTGGGCTCGGAGATGTGTATAAGAGA -3′ 5′-CAGACTCGCTCGCTGCCGGACTACNVGGGTWTCTAAT-3′
EMP16S-10	F:5′-TCGTCGGCAGCGTCAGATGTGTATAAGAGACAGTTGCTGGAC-3′ 5′-GCTGTGTGYCAGCMGCCGCGGTAA-3′
	R:5′-GTCTCGTGGGCTCGGAGATGTGTATAAGAGACAGTTCCTTAGAG-3′ 5′-TCCGGACTACNVGGGTWTCTAAT-3′
EMP16S-11	F:5′-TCGTCGGCAGCGTCAGATGTGTATAAGAGACAGTACTAAC-3′ 5′-GCGGTGTGTGYCAGCMGCCGCGGTAA-3′
	R:5′-GTCTCGTGGGCTCGGAGATGTGTATAAGAGACAGCGTCCG -3′ 5′-TATGAACCGGACTACNVGGGTWTCTAAT-3′
EMP16S-12	F:5′-TCGTCGGCAGCGTCAGATGTGTATAAGAGACAGGCGATCACAC-3 5′-CTGTGTGYCAGCMGCCGCGGTAA-3′
	:5′-GTCTCGTGGGCTCGGAGATGTGTATAAGAGACAGACGTGAGAAC-3′ 5′-GCCGGACTACNVGGGTWTCTAAT-3′
EMP16S-13	F:5′-TCGTCGGCAGCGTCAGATGTGTATAAGAGACAGCAAACGCAC-3′ 5′-TAAGTGTGYCAGCMGCCGCGGTAA-3′
	R:5′-GTCTCGTGGGCTCGGAGATGTGTATAAGAGACAGGGTTGCCCTG-3′ 5′-TACCGGACTACNVGGGTWTCTAAT-3′
EMP16S-14	F:5′-TCGTCGGCAGCGTCAGATGTGTATAAGAGACAGGAGAGGGTT--3′ 5′-GAGTGTGYCAGCMGCCGCGGTAA-3′
	R:5′-GTCTCGTGGGCTCGGAGATGTGTATAAGAGACAGCATA-3′ 5′-TAGCCCGACCGGACTACNVGGGTWTCTAAT-3′
EMP16S-15	F:5′-TCGTCGGCAGCGTCAGATGTGTATAAGAGACAGTGAG-3′ 5′-TGGTCTGTGTGTGYCAGCMGCCGCGGTAA-3′
	R:5′-GTCTCGTGGGCTCGGAGATGTGTATAAGAGACAGGCCTATGA-3′ 5′-GATCCCGGACTACNVGGGTWTCTAAT-3′

**Table 2:** (**a**): The count of selected Gram-positive microbiota during the application experiment (*n* = 12). (**b**): The count of selected Gram-negative microbiota during the application experiment *(n* = 12).

(**a**)					
Sampling	Enterococci	LAB	CoPS	CoNS	Clostridiae
Day 0/1	5.03 ± 1.12 ^b^	7.74 ±1.20 ^b^	5.13 ± 0.93	4.41 ± 0.49	4.25 ± 0.46 ^a^
Day 21	3.65 ± 1.62 ^a^	6.58 ± 0.22 ^a^	4.59 ± 0.80	4.54 ± 0.33	5.44 ± 0.91 ^b^
Day 42	4.38 ± 0.90	7.62 ± 0.41 ^c^	5.3 1 ± 0.43	4.52 ± 0.79	4.89 ± 0.85
Sum *p* value	*p* = 0.0365	*p* = 0.0019	*p* = 0.0842	*p* = 0.8297	*p* = 0.0021
(**b**)					
Sampling	Campylobacter	Pseudomonas	Aeromonas	Coliforms	
Day 0 on	7.63 ± 0.45 ^b^	3.13 ± 0.83	7.24 ± 0.80 ^b^	7.92 ± 0.9 ^b^	
Day 21	3.56 ± 1.83 ^a^	2.46 ± 1.06	5.54 ± 1.37 ^a^	5.10 ± 1.13 ^a^	
Day 42	5.1 0 ± 0.00 ^c^	2.42 ± 0.39	7.00 ± 1.02 ^c^	5.36 ± 0.58 ^c^	
Sum *p* value	*p* < 0.0001	*p* = 0.0657	*p* = 0.0011	*p* < 0.0001	

Enterococci: Day 0/1: Day 21, ^ab^ *p* < 0.05; LAB, lactic acid bacteria, Day 0/1: Day21, ^ab^ *p* < 0.01; Day21: Day 42, ^ac^ *p* < 0.05; CoNS, coagulase-negative staphylococci-NS; CoPS, coagulase-positive staphylococci, NS; *Clostridium*-like, Day 0/1: Day 21, ^ab^ *p* < 0.01; Day 0/1, before PS 412 application; Day 21, after a 3-week application of PS 412; Day 42, after a 3-week cessation of PS 412. *Campylobacter* spp.-like, Day 0/1: Day42, ^ac^ *p* < 0.001; Day 21: Day42, ^cb^ *p* < 0.01; PS, Pseudomonads = NS; *Aeromonas* spp. ^ab^ *p* < 0.01; ^cb^ *p* < 0.01; Coliforms, Day 0/1: Day21, ^ba^ *p* < 0.001; ^bc^ *p* < 0.001; Day 0/1, before PS 412 application; Day 21, after a 3-week application of PS 412; Day 42, after a 3-week cessation of PS 412.

**Table 3:** Microbiota detected using next-generation sequencing at the phyla level in relative abundance (%).

Phyla in % Abundance	Day 0/1	Day 21	Day 42
Bacteroidota	35.38%	45.81%	41.77%
Bacillota	32.25%	28.10%	23.04%
Proteobacteria	13.36%	8.19%	10.83%
Lentisphaerae	10.03%	8.83%	12.23%
Spirochaetota	4.01%	3.34%	3.41%
Fibrobacterota	2.01%	1.32%	5.72%
Euryarcheota	0.65%	1.51%	0.81%
Actinobacteria	0.57%	0.71%	0.29%
Cyanobacteria	0.56%	0.47%	1.09%
Verrumicrobiota	0.54%	0.74%	0.34%
Tenericutes	0.21%	0.16%	0.19%
Synergistota	0.21%	0.51%	0.11%
Saccharibacteria	0.12%	0.16%	0.07%
Planctomycetota	0.06%	0.07%	0.05%
Armatimonadota	0.05%	0.06%	0.03%

**Table 4:** The average value of the phagocytic activity (PA) and index of phagocytic activity (IPA) measured in blood sera during the experiment (in %) and the GPx values in U/gHb (±SD).

Sampling	Day 0/1	Day 21	Day 42	*p* Value
Phagocytic activity	67.89 ± 3.66	69.00 ± 3.91	66.67 ± 2.74	*p* = 0.3762
IPA	1.76 ± 0.75	1.52 ± 0.65	1.93 ± 0.52	
GPx	Day 0/1	Day 21	Day 42	
	156.4 ± 37.51	165.7 ± 64.27	163.6 ± 44.83	*p* = 0.3291

Day 0/1, before application of PS 412; Day 21, after a 3-week application of PS 412; Day 42, after a 3-week cessation of PS 412. GPX, glutathione-peroxidase.

**Table 5:** The values of the enzymatic and hepatic profiles in horses on different sampling days.

**Sampling**	**AST µkat/L**	**ALT µkat/L**	**ALP µkat/L**	**GMT/GGT µkat/L**
Day 0/1	4.23 ± 0.225 ^b^	0.207 ± 0.029	2.09 ± 0.431	0.166 ± 0.042
Day 21	3.82 ± 0.189 ^a^	0.125 ± 0.013	1.99 ± 0.29	0.119 ± 0.021
Day 42	4.18 ± 0.266	0.220 ± 0.049	1.63 ± 0.176	0.181 ± 0.032
Reference value	2.5–5.0	0.06–0.35	0.4–4.0	0.1–0.42
**Sampling**	**Total Bilirubin** **µmol/L**	**CK µkat/L**	**GLDH µkat/L**	**LDH-L µkat/L**
Day 0/1	32.32 ± 3.567	3.82 ± 0.633	0.069 ± 0.015	2.258 ± 0.426 ^c^
Day 21	26.99 ± 1.922	2.60 ± 0.304	0.072 ± 0.010	0.420 ± 0.117 ^b^
Day 42	30.24 ± 2.801	4.02 ± 0.579	0.108 ± 0.025	0.980 ± 0.355 ^a^
Reference value	1.7–39.0	1.0–5.0	up to 0.136	

LDH-L, Day 0/1: Day 42, ^ca^, *p* < 0.05; Day 0/1: Day 21, ^ab^ *p* < 0.001; AST, Day 0/1: Day 21: Day 42, ^ab^ *p* < 0.05; AST, aspartate aminotransferase; ALT, alanine aminotransferase, ALP, alkaline phosphatase; GMT/GGT, ɤ-glutamyl transferase, CK, creatine kinase; GLDH, glutamate dehydrogenase; LDH-L, lactate dehydrogenase; Ca, calcium; P, phosphorus; Mg, magnesium; Day 0/1, before application of PS 412; Day 21, after a 3-week application of PS 412; Day 42, after a 3-week cessation of PS 412; Reference ranges were included in Biochemical testing (Internal Clinic of University of Veterinary Medicine and Pharmacy in Košice (Slovakia).

**Table 6:** The values of the energy profile in horses on different sampling days.

Sampling	Triglycerides mmol/L	HDL Cholesterol mmol/L	LDL Cholesterol mmol/L
Day 0/1	0.910 ± 0.047	1.148 ± 0.036	1.487 ± 0.087 ^b^
Day 21	0.983 ± 0.037	1.016 ± 0.066	1.143 ± 0.048 ^a^
Day 42	1.015 ± 0.032	1.151 ± 0.061	1.294 ± 0.075
Reference value	up to 1.0	1.30–1.41	0.77–0.92

Day 0/1, before application of PS 412; Day 21, after a 3-week application of PS 412; Day 42, after a 3-week cessation of PS 412; Day 0/1: Day 21, ^ab^ *p* < 0.01; Reference ranges were included in Biochemical testing (Internal Clinic of University of Veterinary Medicine and Pharmacy in Košice (Slovakia).

**Table 7:** Nitrogen and mineral profile in horses on different sampling days.

**Sampling**	**Ca mmol/L**	**P mmol/L**	**Mg mmol/L**	**Chlorides**
Day 0/1	2.775 ± 0.078	2.168 ± 0.654 ^a^	1.223 ± 0.046 ^a^	102.5 ± 2.837 ^a^
Day 21	2.516 ± 0.071 ^a^	0.572 ± 0.033 ^b^	1.388 ± 0.089 ^c^	117.8 ± 3.264 ^b^
Day 42	2.385 ± 0.105 ^b^	0.710 ± 0.048 ^c^	0.979 ± 0.039 ^b^	102.3 ± 1.641
Reference value	2.2–3.3	0.7–1.5	0.5–0.9	95–109
**Sampling**	**Total proteins g/L**	**Albumin g/L**	**Urea mmol/L**	**Creatinine µmol/L**
Day 0/1	74.71 ± 4.125	41.50 ± 1.129	3.86 ± 0.590	105.00 ± 10.48
Day 21	68.69 ± 3.164	37.30 ± 1.752	3.83 ± 0.390	113.10 ± 13.93
Day 42	68.41 ± 1.914	38.17 ± 1.104	3.48 ± 0.337	129.30 ± 6.797
Reference value	55–75	35–80	2.5–3.8	71–156

Mineral profile: Ca/21:Ca/42, ^ab^ *p* < 0.05; P0/1:P/21 ^ab^ *p* < 0.01; P/0/1:P/42 ^ac^ *p* < 0.05; Mg/0/1:Mg/42, ^ac^ *p* < 0.001; Mg/42:Mg/21, ^bc^ *p* < 0.001; Chlorides, CH/0/1:CH/21:CH/42, ^ab^ *p* < 0.01; Nitrogen profile, NS; Day 0/1, before application of PS 412; Day 21, after a 3-week application of PS 412; Day 42, after a 3-week cessation of PS 412; Reference ranges were included in Biochemical testing (Internal Clinic of University of Veterinary Medicine and Pharmacy in Košice (Slovakia).

**Table 8:** Blood/hematological values on days 21 and 42 (after PS 412 application and cessation).

Parameter	Reference Value	Day 21	Day 42	*p* Value
White blood cells (WBC)	5.40–14.30	5.77 ± 1.44	6.33 ± 1.54	0.2259
Lymphocyte (LYM)	1.50–7.70	2.198 ± 0.72	2.54 ± 1.18	0.3195
Monocytes (MON)	0.00–1.50	0.20 ± 0.13	0.27 ± 0.11	0.1769
Neutrophils (NEU)	2.30–9.50	3.23 ± 0.89	3.61 ± 0.99	0.3262
Eosinophiles (EOS)	0.10–1.00	0.17 ± 0.03	0.17 ± 0.09	0.9404
Basophiles (BAS)	0.00–0.60	0.01 ± 0.01	0.02 ± 0.01	0.0177
Percent of LYM	17.0–68.0	38.06 ± 9.65	35.25 ± 7.53	0.3498
Percent of MON	0.00–14.00	3.26 ± 1.80	4.51 ± 1.98	0.1274
Percent of NEU	22.00–80.00	55.59 ± 9.74	57. 20 ± 8.13	0.6093
Percent of EOS	1.00–8.00	2.83 ± 1.52	2.69 ± 1.29	0.7061
Percent of BAS	0.00–3.00	0.29 ± 0.00	0.41 ± 0.22	0.0779
Red blood cells (RBC)	6.80–12.90	7.46 ± 0.73	7.70 ± 0.80	0.1101
Hemoglobin, HGB	11.00–19.00	12.04 ± 0.00	12.00 ± 1.27	0.5527
Hematocrit, HCT	32.00–53.00	35.20 ± 4.09	35.30 ± 4.00	0.9136
Medium count of RBC (MCV)	37.00–59.00	47.55 ± 3.08	45.73 ± 2.80	0.0021 **
Thrombocytes PTL	100–400	74.00 ± 26.80	119.8 ± 34.90	0.0023 **

Day 21, after a 3-week application of PS 412; Day 42, after a 3-week cessation of PS 412. Reference values were compared from LABOKLIN actuell s. r.o., Bratislava, Slovakia: Hematology for horse practice. The sampling on day 0/1 was not included. ** *p* < 0.001.

## Data Availability

All data discussed are contained in the article.

## References

[1] Cooke C.G., Gibb Z., Harnett J.E. (2021). The safety, tolerability, and efficacy of probiotic bacteria for equine use. J. Equine Vet. Sci..

[2] Revajová V., Karaffová V., Levkutová M., Šefcová M., Lauková A., Levkut M. (2020). Reaction of immune cells to *Campylobacter jejuni* in chicken PBMC treated by different probiotic bacteria in vitro. Approaches Poult. Dairy Vet. Sci..

[3] Lauková A., Chrastinová Ľ., Pogány Simonová M., Strompfová V., Plachá I., Čobanová K., Formelová Z., Chrenková M., Ondruška Ľ. (2012). *Enterococcus faecium* AL41:Its Enterocin M and their beneficial use in rabbit husbandry. Prob. Antimicro. Prot..

[4] Lauková A., Styková E., Kubašová I., Strompfová V., Gancarčíková S., Plachá I., Miltko R., Belzecki G., Valocký I., Pogány Simonová M. (2020). Enterocin M-producing *Enterococcus faecium* CCM8558 demonstrating probiotic properties in horses. Prob. Antimicro. Prot..

[5] Lauková A., Micenková L., Kubašová I., Bino E., Kandričáková A., Plachá I., Štrkolcová G., Gálik B., Kováčik A., Halo M. (2023). Microbiota, phagocytic activity, biochemical parameters and parasite control in horses with application of autochthonous, bacteriocin-producing, probiotic strain *Enterococcus faecium* EF412. Prob. Antimicro. Prot..

[6] Franz C.H.M.A.P., van Belkum M.J., Holzapfel W.H., Abriouel H., Gálvez A. (2007). Diversity of enterococcal bacteriocins and their grouping in a new classification scheme. FEMS Microbiol. Rev..

[7] Nes I.F., Diep D.B., Moss M.O. (2014). Enterococcal bacteriocins and antimicrobial proteins that contribute to niche control. Enterococci from Commensals to Leading of Drug Resistant Infection.

[8] Lauková A., Styková E., Kubašová I., Gancarčíková S., Plachá I., Mudroňová D., Kandričáková A., Miltko R., Blezecki G., Valocký I. (2018). Enterocin M and its beneficial effects in horses-pilot experiment. Prob. Antimicro. Prot..

[9] Lauková A., Simonová M., Strompfová V., Štyriak I., Ouwehand A.C., Várady M. (2008). Potential of enterococci isolated from horses. Anaerobe.

[10] Salminen S., Collado M.C., Endo A., Hill C., Lebeer S., Quigley E.M.M., Sanders M.E., Shamir R., Swann J.R., Szajewska H. (2021). The International Scientific Association of Probiotics and Prebiotics (ISAPP) consensus on the definition and scope of postbiotics. Nat. Rev. Gastroenterol. Hepatol..

[11] Chikindas M.L., Sichel L.S., Popov I.V., Tag J.R., Lu X., Mitrokhin O.V., Todorov S.D. (2025). Postbiotics: What are they?. Ben. Microbes.

[12] Liang B., Xing D. (2023). The current and future perspectives of postbiotics. Prob. Antimicro. Prot..

[13] Mosca A., Abreu YAbreu A.T., Gwee K.A., Ianiro G., Tack J., Nguyen T.V.H., Hill C. (2022). The clinical evidence for postbiotics as microbial therapeutics. Gut Microbes.

[14] Mareková M., Lauková A., De Vuyst L., Skaugen M., Nes I.F. (2003). Partial characterization of bacteriocins produced by environmental strain *Enterococcus faecium* EK13. J. Appl. Microbiol..

[15] De Vuyst L., Callewaert R., Pot B. (1996). Characterization of the antagonistic activity of *Lactobacillus amylovorus* DCE471 and large- scale isolation of its bacteriocin amylovorin L471. Syst. Appl. Microbiol..

[16] Aronesty E. (2011). ea-utils. Command-Line Tools for Processing Biological Sequencing Data. https://github.com/ExpressionAnalysis/ea-utils.

[17] Bolger A.M., Lohse Usadel B. (2014). Trimmomatic: A flexible trimmer for Illumina sequence data. Bioinformatics.

[18] Edgar R.C. (2010). Search and clustering orders of magnitude faster than BLAST. Bioinformatics.

[19] Caporaso J.G., Kuczynski J., Stombaugh J., Bittinger K., Bushman F.D., Costello E.K., Fierer N., Peña A.G., Goodrich J.K., Gordon J.I. (2010). QUIME allows analysis of high-throughput community sequencing data. Nat. Meth..

[20] Quast C., Pruesse E., Yilmaz P., Gerken J., Schweer T., Yarza P., Peplies J., Glöckner F.O. (2013). SILVA ribosomal RNA gene database project: Improved data processing and web-based tools. Nucleic Acids Res..

[21] Oren A., Garrity G.M. (2021). Valid publication of the names of forty-two phyla of prokaryotes. Int. J. Syst. Evol. Microbiol..

[22] Kolesárová A., Capcarová M., Arpášová H., Kalafová A., Massányi P., Lukáč N., Kováčik A., Schneidgenová M. (2008). Nickel-induced blood biochemistry alterations in hens after an experimental per oral administration. Environ. Sci. Health.

[23] Kováčik A., Arvay J., Tušimová E., Harangozó Ľ., Tvrdá E., Zbynovská K., Cupka P., Andrašovská Š., Tomas J., Massányi P. (2017). Seasonal variations in the blood concentration of selected heavy metals in sheep and their effects on the biochemical and hematological parameters. Chemosphere.

[24] Divsalar E., İncili G.K., Shi C., Semsari E., Hosseini S.H., Ebrahimi Tirtashi F., Toker O.S., Mojgani N., Liu S.-Q., Moradi M. (2026). Challenges with the use of postbiotics/parabiotics in food industry. Crit. Rev. Food Sci. Nutr..

[25] Cicenia A., Santangelo F., Gambardella L., Pallotta V., Iebba A., Scirocco A., Marignani M., Tellan G., Carabotti M., Corazziari E.S. (2016). Protective role of postbiotic mediators secreted by *Lactobacillus rhamnosus* GG versus lipopolysaccharide-induced damage in human colonic smooth muscle cells. J. Clin. Gastroenterol..

[26] Dougal K., Harris A.P., Girdwood S.E., Creevey C.H.J., Curtis G.C., Barfoot C.F., Argo C.M., Newbold C.H.J. (2017). Changes in the total fecal bacterial population in individual horses maintained on a restricted diet over 6 weeks. Front. Microbiol..

[27] Piskoríková M. Quality and characterization of existing and new probiotics (EFSA QPS). Proceedings of the Regulatory Framework Workshop Health: Claim Approval of Probiotics in the European Union, Issues, Barriers, Success Drivers.

[28] Gálik B., Bíro D., Halo M., Juráček M., Šimko M., Massányi P., Rolinec M. (2012). The effect of different macromineral intakes on mineral metabolism of sport horses. Acta Vet. Brno.

[29] Hisaeda K., Ono T., Shimokawa-Miyma T., Hata A., Iwata E., Hiasa Y., Ohzawa E., Tozaki T., Murase H., Takasu M. (2025). Differences in serum iron concentrations between the summer and winter in Noma horses. J. Equine Sci..

[30] Terpeluk E.R., Schafer J., Finkler-Schade C., Schuberth H.J. (2024). Supplementation of Foals with a *Saccharomyces cerevisiae* Fermentation Product Alters the Early Response to Vaccination. Animals.

[31] Lucassen A., Finkler-Schade C., Schuberth H.J. (2021). A *Saccharomyces cerevisiae* Fermentation Product (Olimond BB) Alters the Early Response after Influenza Vaccination in Racehorses. Animals.

[32] Kraft S., Buchenauer L., Polte T. (2021). Mold, Mycotoxins and a Dysregulated Immune System: A Combination of Concern?. Int. J. Mol. Sci..

[33] Sahdeo P., Bhaumik P., Prafulla K., Rajiv L. (2025). Postbiotics: Multifunctional Microbial products transforming animal health and performance. Vet. Sci..

